# Supplementary material for: HIVprotI: an integrated web based platform for prediction and design of HIV proteins inhibitors
Source: J Cheminform. 2018 Mar 9;10:12. doi: 10.1186/s13321-018-0266-y (PMC5845081; doi:10.1186/s13321-018-0266-y)
Supplement: Supplementary file 2 — Additional file 2. Source code of HIVProtI web server. [file 13321_2018_266_MOESM2_ESM.zip › HIVprotI_Source-code/hivprotI/weka-3-6-6/doc/weka/core/FastVector.html]

FastVector


---


|  |  |  |
| --- | --- | --- |
| **Overview** \| **Package** \| **Class** \| **Tree** \| **Deprecated** \| **Index** \| **Help** \| **Weka's home** | |  |
| **PREV CLASS**   **NEXT CLASS** | **FRAMES**    **NO FRAMES**     **All Classes** |  |
| SUMMARY: NESTED \| FIELD \| CONSTR \| METHOD | DETAIL: FIELD \| CONSTR \| METHOD |  |


---


## weka.core Class FastVector

```
java.lang.Object
  weka.core.FastVector
```

**All Implemented Interfaces:**: java.io.Serializable, Copyable, RevisionHandler

**Direct Known Subclasses:**: BestFirst.LinkedList2, LFSMethods.LinkedList2

---

``` public class FastVector extends java.lang.Object implements Copyable, java.io.Serializable, RevisionHandler ```

Implements a fast vector class without synchronized
methods. Replaces java.util.Vector. (Synchronized methods tend to
be slow.)

**Version:**
:   $Revision: 1.16 $

**Author:**
:   Eibe Frank (eibe@cs.waikato.ac.nz)

**See Also:**: Serialized Form

---

| **Nested Class Summary** | |
| --- | --- |
| `class` | `FastVector.FastVectorEnumeration`             Class for enumerating the vector's elements. |


**Constructor Summary**

`FastVector()`
  
          Constructs an empty vector with initial
capacity zero.

`FastVector(int capacity)`
  
          Constructs a vector with the given capacity.


| **Method Summary** | |
| --- | --- |
| `void` | `addElement(java.lang.Object element)`             Adds an element to this vector. |
| `void` | `appendElements(FastVector toAppend)`             Appends all elements of the supplied vector to this vector. |
| `int` | `capacity()`             Returns the capacity of the vector. |
| `boolean` | `contains(java.lang.Object o)`             added by akibriya |
| `java.lang.Object` | `copy()`             Produces a shallow copy of this vector. |
| `java.lang.Object` | `copyElements()`             Clones the vector and shallow copies all its elements. |
| `java.lang.Object` | `elementAt(int index)`             Returns the element at the given position. |
| `java.util.Enumeration` | `elements()`             Returns an enumeration of this vector. |
| `java.util.Enumeration` | `elements(int index)`             Returns an enumeration of this vector, skipping the element with the given index. |
| `java.lang.Object` | `firstElement()`             Returns the first element of the vector. |
| `java.lang.String` | `getRevision()`             Returns the revision string. |
| `int` | `indexOf(java.lang.Object element)`             Searches for the first occurence of the given argument, testing for equality using the equals method. |
| `void` | `insertElementAt(java.lang.Object element, int index)`             Inserts an element at the given position. |
| `java.lang.Object` | `lastElement()`             Returns the last element of the vector. |
| `void` | `removeAllElements()`             Removes all components from this vector and sets its size to zero. |
| `void` | `removeElementAt(int index)`             Deletes an element from this vector. |
| `void` | `setCapacity(int capacity)`             Sets the vector's capacity to the given value. |
| `void` | `setElementAt(java.lang.Object element, int index)`             Sets the element at the given index. |
| `int` | `size()`             Returns the vector's current size. |
| `void` | `swap(int first, int second)`             Swaps two elements in the vector. |
| `java.lang.Object[]` | `toArray()`             Returns all the elements of this vector as an array |
| `void` | `trimToSize()`             Sets the vector's capacity to its size. |

**Methods inherited from class java.lang.Object**

`equals, getClass, hashCode, notify, notifyAll, toString, wait, wait, wait`

**Constructor Detail**

### FastVector

```
public FastVector()
```

:   Constructs an empty vector with initial
    capacity zero.

---


### FastVector

```
public FastVector(int capacity)
```

:   Constructs a vector with the given capacity.

    **Parameters:**: `capacity` - the vector's initial capacity


**Method Detail**

### addElement

```
public final void addElement(java.lang.Object element)
```

:   Adds an element to this vector. Increases its
    capacity if its not large enough.

    :   **Parameters:**: `element` - the element to add

---


### capacity

```
public final int capacity()
```

:   Returns the capacity of the vector.

    :   **Returns:**: the capacity of the vector

---


### copy

```
public final java.lang.Object copy()
```

:   Produces a shallow copy of this vector.

    :   **Specified by:**: `copy` in interface `Copyable`
    :   **Returns:**: the new vector

---


### copyElements

```
public final java.lang.Object copyElements()
```

:   Clones the vector and shallow copies all its elements.
    The elements have to implement the Copyable interface.

    :   **Returns:**: the new vector

---


### elementAt

```
public final java.lang.Object elementAt(int index)
```

:   Returns the element at the given position.

    :   **Parameters:**: `index` - the element's index **Returns:**: the element with the given index

---


### elements

```
public final java.util.Enumeration elements()
```

:   Returns an enumeration of this vector.

    :   **Returns:**: an enumeration of this vector

---


### elements

```
public final java.util.Enumeration elements(int index)
```

:   Returns an enumeration of this vector, skipping the
    element with the given index.

    :   **Parameters:**: `index` - the element to skip **Returns:**: an enumeration of this vector

---


### contains

```
public boolean contains(java.lang.Object o)
```

:   added by akibriya

---


### firstElement

```
public final java.lang.Object firstElement()
```

:   Returns the first element of the vector.

    :   **Returns:**: the first element of the vector

---


### indexOf

```
public final int indexOf(java.lang.Object element)
```

:   Searches for the first occurence of the given argument,
    testing for equality using the equals method.

    :   **Parameters:**: `element` - the element to be found **Returns:**: the index of the first occurrence of the argument in this vector; returns -1 if the object is not found

---


### insertElementAt

```
public final void insertElementAt(java.lang.Object element,
                                  int index)
```

:   Inserts an element at the given position.

    :   **Parameters:**: `element` - the element to be inserted: `index` - the element's index

---


### lastElement

```
public final java.lang.Object lastElement()
```

:   Returns the last element of the vector.

    :   **Returns:**: the last element of the vector

---


### removeElementAt

```
public final void removeElementAt(int index)
```

:   Deletes an element from this vector.

    :   **Parameters:**: `index` - the index of the element to be deleted

---


### removeAllElements

```
public final void removeAllElements()
```

:   Removes all components from this vector and sets its
    size to zero.

---


### appendElements

```
public final void appendElements(FastVector toAppend)
```

:   Appends all elements of the supplied vector to this vector.

    :   **Parameters:**: `toAppend` - the FastVector containing elements to append.

---


### toArray

```
public final java.lang.Object[] toArray()
```

:   Returns all the elements of this vector as an array

    :   **Returns:**: an array containing all the elements of this vector

---


### setCapacity

```
public final void setCapacity(int capacity)
```

:   Sets the vector's capacity to the given value.

    :   **Parameters:**: `capacity` - the new capacity

---


### setElementAt

```
public final void setElementAt(java.lang.Object element,
                               int index)
```

:   Sets the element at the given index.

    :   **Parameters:**: `element` - the element to be put into the vector: `index` - the index at which the element is to be placed

---


### size

```
public final int size()
```

:   Returns the vector's current size.

    :   **Returns:**: the vector's current size

---


### swap

```
public final void swap(int first,
                       int second)
```

:   Swaps two elements in the vector.

    :   **Parameters:**: `first` - index of the first element: `second` - index of the second element

---


### trimToSize

```
public final void trimToSize()
```

:   Sets the vector's capacity to its size.

---


### getRevision

```
public java.lang.String getRevision()
```

:   Returns the revision string.

    :   **Specified by:**: `getRevision` in interface `RevisionHandler`
    :   **Returns:**: the revision


---


|  |  |  |
| --- | --- | --- |
| **Overview** \| **Package** \| **Class** \| **Tree** \| **Deprecated** \| **Index** \| **Help** \| **Weka's home** | |  |
| **PREV CLASS**   **NEXT CLASS** | **FRAMES**    **NO FRAMES**     **All Classes** |  |
| SUMMARY: NESTED \| FIELD \| CONSTR \| METHOD | DETAIL: FIELD \| CONSTR \| METHOD |  |


---
